# Lipidic Cubic-Phase Nanoparticles (Cubosomes) Loaded with Doxorubicin and Labeled with ^177^Lu as a Potential Tool for Combined Chemo and Internal Radiotherapy for Cancers

**DOI:** 10.3390/nano10112272

**Published:** 2020-11-16

**Authors:** Adrianna Cytryniak, Ewa Nazaruk, Renata Bilewicz, Emilia Górzyńska, Kinga Żelechowska-Matysiak, Rafał Walczak, Adam Mames, Aleksander Bilewicz, Agnieszka Majkowska-Pilip

**Affiliations:** 1 Faculty of Chemistry, University of Warsaw, Pasteura 1 St., 02-093 Warsaw, Poland; acytryniak@chem.uw.edu.pl (A.C.); enaz@chem.uw.edu.pl (E.N.); bilewicz@chem.uw.edu.pl (R.B.); 2 Centre of Radiochemistry and Nuclear Chemistry, Institute of Nuclear Chemistry and Technology, Dorodna 16 St., 03-195 Warsaw, Poland; e.gorzynska@student.uw.edu.pl (E.G.); k.zelechowska@ichtj.waw.pl (K.Ż.-M.); r.walczak@ichtj.waw.pl (R.W.); a.bilewicz@ichtj.waw.pl (A.B.); 3 Institute of Physical Chemistry, Polish Academy of Sciences, Kasprzaka 44/52 St., 01-224 Warsaw, Poland; amames@ichf.edu.pl

**Keywords:** cubosome, ^177^Lu, doxorubicin, chemotherapy, radiotherapy, combined anticancer therapy, drug carrier

## Abstract

Lipid liquid-crystalline nanoparticles (cubosomes) were used for the first time as a dual-modality drug delivery system for internal radiotherapy combined with chemotherapy. Monoolein (GMO)-based cubosomes were prepared by loading the anticancer drug, doxorubicin and a commonly used radionuclide, low-energy beta (β^−^)-emitter, ^177^Lu. The radionuclide was complexed with a long chain derivative of DOTAGA (DOTAGA-OA). The DOTAGA headgroup of the chelator was exposed to the aqueous channels of the cubosomes, while, concerning OA, the hydrophobic tail was embedded in the nonpolar region of the lipid bilayer matrix, placing the radioactive dopant in a stable manner inside the cubosome. The cubosomes containing doxorubicin and the radionuclide complex increased the cytotoxicity measured by the viability of the treated HeLa cells compared with the effect of single-drug cubosomes containing either the DOX DOTAGA-OA or DOTAGA-OA-^177^Lu complex. Multifunctional lipidic nanoparticles encapsulating the chemotherapeutic agent together with appropriately complexed (β^−^) radionuclide are proposed as a potential strategy for effective local therapy of various cancers.

## 1. Introduction

The most common method for cancer treatment is currently chemotherapy. Among the many chemotherapeutic agents, doxorubicin (DOX) is an effective drug applied to treat both solid tumors (breast cancer, carcinomas) and hematologic malignancies (leukemia, lymphomas). The extensive usage of DOX for over 50 years has substantially improved cancer survival statistics; however, the very low tumor selectivity of systemic chemotherapy using DOX and its serious side-effects limit the applications of this therapy. A consequence of these side-effects is the application of suboptimal doses, which often results in therapeutic failure and development of drug resistance. As a result, dose-dependent cardiotoxicity compromises its clinical usage [1,2]. 

Besides chemotherapy, radiation therapy is an important method for the treatment of tumors and is applied for approximately 50% of all cancer patients [3]. The crucial point in cancer radiotherapy is achieving a high therapeutic efficacy by maximizing damage to the tumor while sparing healthy tissues. To increase the treatment effectiveness, the combination of external radiotherapy and chemotherapy has recently been incorporated into medical practices. The synergistic effect of chemotherapy and radiation therapy is already known [4,5,6]; it is mainly due to the sensitizing effect of ionizing radiation on the chemotherapy agents which allows to use much lower chemotherapeutic dosages, and is important especially in the case of highly cardiotoxic drugs such as doxorubicin.

The extension of radiation therapy based on the external radiation source is the internal radiotherapy, where the radionuclides linked to a targeting vector are administered. This can be achieved by conjugating suitable radionuclides with antibodies, antibody fragments, nanobodies, or small peptides which allows them to bind to the receptors at the cell surfaces or to other proteins highly expressed in cancer cells.

In the past 10 years, nanomedicine employing multi-functional nanomaterials has been proposed to combine radiotherapy and chemotherapy. The development of biocompatible/biodegradable nano-platforms with multiple functionalities for chemo-radioisotope therapy may have significant potential for clinical treatment of cancers. Few publications have described in vitro and in vivo studies of liposomes that contain both chemotherapeutics and β^−^emitters. Gao et al. [7] showed that the combination therapy increased the survival rates of mice with small tumors compared to the monotherapy. In another approach, Chen et al. used ^188^Re and doxorubicin-encapsulated liposomes for colorectal adenocarcinoma (HT-29 cells) therapy [8]. Without any targeting molecules. it exhibited a high tumor/background ratio. Analogous radiolabeled liposomes have been investigated on other cancer models [9,10,11,12]. Targeted therapy, based on the folate-functionalized lipid nanoparticle containing doxorubicin and ^90^Y radionuclide, was studied on a carcinoma cell line CNE1 and showed significant tumor growth suppression compared with the control groups. [13].

Lipidic liquid-crystalline phases (LCP), such as cubic phases, are promising drug carriers. They have large interfacial areas of 400 m^2^/g to anchor the drugs, and their structures consist of a bicontinuous lipid bilayer surrounding two systems of non-contacting aqueous channels [14]. A wide range of drugs can be encapsulated within this biocompatible amphiphilic environment, which resembles a biological membrane [15,16,17,18,19]. Cubic phases are stable in the presence of excess of water and can be dispersed in the presence of a stabilizer into kinetically stable colloidal nanoparticles, or cubosomes. Cubosomes are less viscous and exist as a suspension, which makes them easier to handle and deliver to the appropriate site. Considering this, the cubosomes may become useful as drug delivery systems for local chemotherapy [20,21]. Among lipid nanoparticles, they have been most extensively studied because of their sustained drug release properties and ability to maintain a three-dimensional structure under a range of physiologically relevant conditions. Compared with liposomes, cubosomes have a larger breaking resistance and a larger ratio of bilayer area to the particle volume. Cubosomes have been already proven to be excellent drug delivery systems [22] used also for anticancer drugs [23,24] and for transport of drugs across the blood–brain barrier [25]. When conjugated with specific ligands, e.g., folic acid, they address tumor tissues that overexpress folate receptors and increase the drug delivery efficiency [26,27]. When doped with imaging and therapeutic agents, they can be beneficial for theranostic nanomedicine [28,29]. Co-delivery of two drugs loaded into cubosomes has been recently reported [30,31]. Dual-modality cubosomes were used for fluorescence-MR imaging [32,33]. When loaded with an anticancer drug and NIR fluorescent probe, they showed increased targeting ability toward HeLa cancer cells [29]. Monoolein is the most common lipid used to prepare cubosomal drug delivery systems [34,35,36,37,38,39].

**Figure 1:**
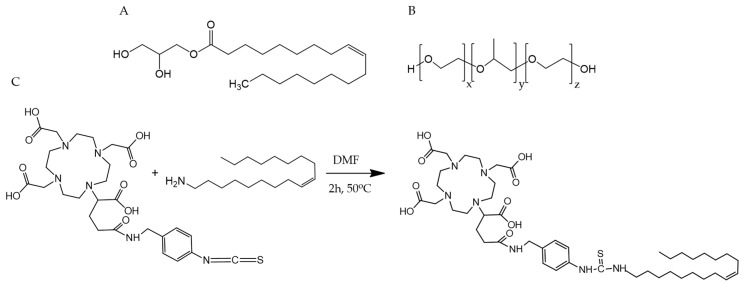
(**A**) Cubosome forming lipid, monoolein (GMO), (**B**) cubosome stabilizer, Pluronic F-127, and (**C**) synthetic scheme for cubosome dopant, DOTAGA-OA conjugate formation.

Due to the previously demonstrated excellent properties of cubosomes [34] as drug carriers, we decided to apply GMO-based cubosomes (Figure 1A,B) as a carrier of both a radionuclide and chemotherapeutic drug. Based on our previous work [26,34], we selected the most widely used doxorubicin as the chemotherapeutic, and as the radionuclide—the common low-energy β-emitter, ^177^Lu, exhibiting suitable nuclear properties for nuclear medicine applications (*T*_1/2_ = 6.7 d; *E***_β_**_(max)_ = 0.497 MeV; maximum soft-tissue penetration of 2 mm). Cubosomes were doped with DOTAGA-oleylamine conjugate (DOTAGA-OA, Figure 1C), which is a chelator forming stable complexes with lutetium-177. By design, the DOTAGA head group was exposed to aqueous channels, whereas the hydrophobic tail was anchored in the lipid bilayer of the cubosomes. For structural and size characterization of cubosomes, small-angle X-ray scattering (SAXS), dynamic light scattering (DLS), and cryogenic transmission electron microscopy (cryo-TEM) were employed. To the best of our knowledge, labeling cubosomes with therapeutic radionuclides or therapeutic radionuclides loaded together with chemotherapeutic agents in the cubosomes has not been reported before.

## 2. Materials and Methods

### 2.1. Radionuclides

Lutetium-177 radionuclide in carrier form of ^177^LuCl_3_ in 0.04 M HCl solution was obtained from Radioisotope Centre Polatom, Świerk, Poland (specific activity > 370 GBq/mg of Lu, radionuclide purity > 99.9%).

### 2.2. Reagents

*p*-NCS-benzyl-DOTA-GA (2,2′,2”-(10-(1-carboxy-4-((4-isothiocyanatobenzyl)amino)-4-oxobutyl)-1,4,7,10-tetraazacyclododecane-1,4,7-triyl) triacetic acid) was purchased from CheMatech (Dijon, France), oleylamine from VWR (Gdańsk, Poland), and dry DMF (*N*,*N*-dimethylformamide) from Sigma-Aldrich (St. Louis, MO, USA).

All deuterated solvents were purchased from Sigma-Aldrich (St. Louis, MO, USA), and used without any further purification. The NMR samples were prepared as follows: 0.5 mL of deuterated solvent and 2 mg of substrate were injected into a 5 mm NMR tube (high-precision WILMAD). 

For ITLC (instant thin layer chromatography), analysis glass microfiber sheets (Agilent, Santa Clara, CA, USA) were used.

Serum aliquots were isolated from blood samples of healthy volunteers and stored at −20 °C. The volunteers were informed about the experimental protocol, and informed consent was obtained. 

The following materials were used for cell experiments: RPMI-1640 Medium, fetal bovine serum, phosphate-buffered saline (PBS), trypsin-EDTA, and penicillin/streptomycin solutions from Biological Industries (Beth Haemek, Israel), dimethyl sulfoxide (DMSO) (Sigma-Aldrich, St. Louis, MO, USA), and CellTiter 96^®^ AQueous One Solution Reagent (MTS compound) from Promega (Madison, WI, USA). Human-derived HeLa cancer cells were obtained from the American Type Culture Collection (ATCC, Rockville, MD, USA) and were cultured in RPMI-1640 Medium supplemented with 10% fetal bovine serum and 1% penicillin/streptomycin. The cells were incubated at 37 °C in a humidified atmosphere containing 5% CO_2_.

Monoolein (1-oleoyl-*rac*-glycerol) purity 99% (GMO), doxorubicin hydrochloride (DOX), and Pluronic^®^ F-127 were used to self-assemble the cubosomes and were purchased from Sigma-Aldrich (St. Louis, MO, USA). Sodium acetate and acetic acid for preparing the buffer solution (0.1 M, pH 5.0) were purchased from Sigma-Aldrich (St. Louis, MO, USA). All solutions were prepared with Milli-Q water (18.2 MΩ cm^−1^; Millipore).

### 2.3. Instrumentation

NMR spectra were acquired on a BRUKER AVANCE II spectrometer equipped with a BBI probe head and BTV temperature controller. ITLC strips were measured using a Cyclone Plus Storage Phosphor System (Perkin-Elmer Life and Analytical Sciences, Shelton, CT, USA) and analyzed by Optiquant software (version 5.0, Waltham, MA, USA). MTS assays were performed using an Apollo 11LB913 microplate reader (Berthold, Bad Wildbad, Germany). 

Small-angle X-ray scattering (SAXS) was used to investigate the structure of cubosome dispersions. SAXS measurements were performed on a Bruker Nanostar system equipped with a Vantec 2000 area detector (Madison, WI, USA), operating with Cu Kα radiation. Two-dimensional patterns were integrated into 1D scattering functions *I*(*q*) (where *q* (nm^−1^) is the length of the scattering vector). The scattering vector *q* was determined from the scattering angle using the relationship *q* = (4*π*/*λ*)sin*θ*, where 2*θ* is the scattering angle, and *λ* is the wavelength of radiation. Before measurements, samples were loaded into 1.5 mm capillaries sealed with epoxy glue (UHU). Measurements were performed at 25 °C and 37 °C and the scattering intensities were collected over a period of 3h for dispersed systems. The Equation (1) was used to calculate the lattice parameter (*a*):

(1) $$a=\frac{2\pi }{q}\times \sqrt{h^{2}+k^{2}+l^{2}}$$

where *q* is the scattering vector; *h*, *k*, *l*—Miller indices of the Bragg peak.

The average size, polydispersity, and zeta potential (*ζ*) of cubosomes were determined using dynamic light scattering (DLS, Zetasizer Nano ZS Malvern, UK) at 25 °C, assuming the viscosity of pure water and presented as an average of three separate measurements. 

Cubosome dispersions were plunge-frozen onto Quantifoil R2/2 holey carbon grids using a Thermo Fisher Vitrobot. Two-dimensional electron cryomicroscopy images were taken on a Thermo Fisher Glacios TEM operating at 200 kV, equipped with a 4k × 4k Falcon 3EC direct electron detection camera at a magnification of 92 k, which corresponds to a pixel size of 1.5 Å at the specimen level. The defocus was set to 2 and 5 μm, and the total electron dose was approximately 50 e/Å^2^.

### 2.4. Synthesis of p-NCS-benzyl-DOTA-GA-Oleylamine Conjugate (DOTAGA-OA)

Oleylamine (5.28 µL, 16.06 μmol) was added to a solution of *p*-NCS-benzyl-DOTAGA (10 mg, 16.06 μmol) in 1.25 dry DMF (purity ≥ 99%), and the reaction mixture was stirred overnight at 50 °C under an inert atmosphere. Subsequently, solvents were removed under reduced pressure. The product was dried under a high vacuum pump for 12 h; 11.9 mg (13.33 μmol, 83%) of *p*-NCS-benzyl-DOTAGA-oleylamine was obtained as a white powder; ^1^H NMR (300 MHz, DMSO-*d*_6_) δ 8.53 (t, *J* = 5.9 Hz, 1H), 7.95 (s, 1H, amide), 7.38 (d, *J* = 8/6 Hz, 2H aryl), 7.31 (d, *J* = 8.6 Hz, 2H, aryl), 5.36–5.25 (m, 2H, CH=CH), 4.26 (d, *J* = 6.00 Hz, 2H, Ar-CH_2_), 3.92–2.90 (m, 8H), 2.82–2.62 (m, 4H), 2.43–2.23 (m, 2H), 1.98 (dd, *J* = 12.6, 6.2 Hz, 4H), 1.54 (dd, *J* = 14.5, 7.1 Hz, 2H), 1.33–1.21 (m, 24H -CH_2_-), 0.87 (t, *J* = 6.50 Hz, 3H, CH_3_); ^13^C NMR (75 MHz, DMSO-*d*_6_) δ: 172.94, 163.56, 141.09, 134.33, 131.34, 130.91, 130.87, 129.83, 129.59, 127.10, 116.36, 54.93, 32.52, 30.33, 30.29, 30.07, 29.98, 29.92, 29.83, 29.79, 28.20, 27.82, 27.10, 23.34, and 15.02. ESI-MS: m/z = 890.6 [M-H]^+^.

### 2.5. Lipophilicity of DOTAGA-OA-^177^Lu Complex

The partition coefficient of the DOTAGA-OA-^177^Lu complex was measured in a biphasic system of *n*-octanol/PBS (pH 7.4). After labeling DOTAGA-OA (34 nmol) with 4.5 MBq of ^177^Lu in acetate buffer (0.1 M, pH 5.5), the radioconjugate was transferred to the prepared biphasic mixture, vortexed for 1 min, and then centrifuged (14,000 rpm, 5 min) [40]. The phases were separated, and the activity of each phase was determined by measuring the radiation in a NaI(Tl) scintillation detector. The partition coefficient, *P*, was calculated as the ratio of radioactivities of the organic and aqueous phases (at least three independent measurements), and the lipophilicity was determined as the logarithm of partition coefficients (log*P*).

### 2.6. Preparation of Cubosomes Loaded with DOTAGA-OA and DOX 

To produce cubosomes, a top-down approach was employed according to a previously described procedure [34]. Cubosomes were prepared by hydrating a lipid in the presence of acetate buffer (0.1 M, pH 5.0) and Pluronic F-127 stabilizer. Then, the sample was sonicated for 20 min using a Sonic 0.5 ultrasonic bath (Polsonic, Poland). To prepare DOTAGA-OA and DOX-doped cubosomes, DOTAGA-OA was first dissolved in a chloroform:methanol mixture (4:1 *v*/*v*). Solvents were evaporated, and DOTAGA-OA (5.7 µmol) and DOX (0.4 µmol) were mixed with the melted lipid (143.4 µmol) before the hydration with Pluronic F-127 (0.5 µmol mL^−1^ in acetate buffer). The amount of DOTAGA-OA in the cubosome formulation was chosen based on radiolabeling conditions. As shown in our previous paper, at acidic pH, DOX is located mainly in the aqueous channels [41]. In the case of DOTAGA-OA, the DOTAGA head group is expected to be exposed to aqueous channels, while the hydrophobic oleylamine tail is embedded in the lipid bilayer domain of the cubosome. Cubosomal formulations were equilibrated at room temperature for at least 24 h. The final compositions of the cubosomes are presented in Table 1.

Free DOX was separated from the obtained cubosomes using centrifugation method. Cubosome formulations were placed in Amicon tubes (Amicon^®^ Ultra 0.5 mL Centrifugal Filters, Sigma Aldrich (St. Louis, MO, USA) and centrifuged at 3000× *g* rpm for approximately 15 min. The ultrafiltrate was then separated and the amount of the unbound DOX was determined using a UV-Vis spectrophotometer (Cary 60, Agilent) at *λ* = 485 nm. The encapsulation efficiency (EE (%)) was calculated using Equation (2):

(2) $$\mathrm{EE}\;\left[\%\right]=\frac{C\left({\mathrm{DOX}}_{\mathrm{TOTAL}}\right)-C\left({\mathrm{DOX}}_{\mathrm{FREE}}\right)}{C\left({\mathrm{DOX}}_{\mathrm{TOTAL}}\right)}\times 100\%$$

where C(DOX_TOTAL_) is the concentration of DOX in cubosomes and C(DOX_FREE_) is the DOX concentration found in the supernatant solution.

### 2.7. Radiolabeling of Cubosomes Loaded with DOX and DOTAGA-OA 

For labeling, 56.8 µL (75 MBq) of ^177^LuCl_3_ in 0.04 M HCl was added to 5.3 µL of cubosome formulation containing 30 nmol DOTAGA-OA or DOTAGA-OA and DOX. Together with 200 µL of acetate buffer (0.1 M, pH 5.0) samples were placed in Eppendorf Tubes^®^. The solution was heated at 95 °C for 30 min, and cooled to room temperature. The labeling efficiency was determined by instant thin-layer chromatography (ITLC) with citrate buffer (0.5 M, pH 5.5) as the mobile phase. In this method, free ^177^Lu moves with the solvent front while labeled conjugates remain at the baseline. The radioactivity distribution on the ITLC strips was measured by a Cyclone Plus Storage Phosphor System and analyzed using Optiquant software supplied by the manufacturer.

### 2.8. Stability Studies of Cubosomes with ^177^Lu 

DOTAGA-OA-^177^Lu or DOX DOTAGA-OA-^177^Lu cubosome dispersion (20 µL) was added to 200 µL of human serum or 200 µL of PBS buffer and incubated at 37 °C. Stability studies were performed after 24 h on three parallel samples using ITLC with citrate buffer as the mobile phase.

### 2.9. Cytotoxicity Evaluation

Cytotoxicity studies were performed for cubosome dispersions containing DOTAGA-OA-^177^Lu, DOX DOTAGA-OA-^177^Lu, and DOTAGA-OA (MO concentration: 54.38 µg/mL), DOX DOTAGA-OA (DOX concentration: 0.21 µg/mL) as cold controls. HeLa cells were seeded in 96-well plates at a density of 2 × 10^3^ cells per well at 37 °C with 5% CO_2_ in a humidified environment. After 24 h, cells were washed with PBS and various doses of radioconjugates (2.5–15 MBq/mL), as well as 100 µL DOTAGA-OA and DOX DOTAGA-OA compounds were added. Treated cells were incubated for an additional 24 h, 48 h, and 72 h. The MTS assays were performed using CellTiter-96^®^ AQueous-Non-Radioactive Cell Proliferation Assay. The absorbance of the formazan in wells was measured at 490 nm using a microplate reader. Results are expressed as the percentage of viable cells relative to the control groups (cells grown in medium only).

### 2.10. Statistical Analysis

All experiments were performed at least in triplicate and repeated three times. For statistical analysis, GraphPad Prism software v. 8.0 (GraphPad Software Inc., San Diego, CA, USA) was used. To evaluate the cytotoxicity, values between groups were compared using two-way ANOVA (followed by Dunnett’s multiple analysis). The results are reported as mean ± standard error of the mean (SEM) and were considered as statistically significant when *p* ≤ 0.05, *p* ≤ 0.01, *p* ≤ 0.001, and *p* ≤ 0.0001.

## 3. Results and Discussion

### 3.1. Synthesis of DOTAGA-OA Conjugate

While cubosomes have been frequently used as drug carriers, they have never been employed as carriers for therapeutic radionuclides. Thus, we decided to apply a complex of the low energy β-emitter ^177^Lu and DOTAGA ligand. DOTAGA metal complexes are hydrophilic and are released very fast from cubic-phase channels; therefore, to anchor them in the lipidic part of the cubic phase, we had to synthesize DOTAGA-OA, a lipophilic conjugate, using an oleylamine hydrophobic chain. The synthesis was performed by reacting bifunctional ligand *p*-NCS-benzyl-DOTAGA, which contains an NCS group with oleylamine (Figure 1C). The final product was obtained in good yield and the identity was confirmed by ^1^H NMR, ^11^C NMR spectroscopy, and MS spectrometry (Supplementary Material Figures S1–S3). The lipophilicity was measured using the partition coefficient between water and *n*-octanol (log*P* = −0.84 ± 0.14) was used to indicate the possibility of easily incorporating DOTAGA-OA conjugate into the cubic-phase. 

To optimize the labeling efficiency of DOTAGA-OA-^177^Lu radioconjugate, various amounts of DOTAGA-OA (5 nmol, 10 nmol, 15 nmol, 20 nmol, 30 nmol) were labeled with 5 MBq ^177^Lu. The labeling yield determined for each sample was >99%; 30 nmol of DOTAGA-OA labeled with 75 MBq ^177^Lu was used in subsequent studies. The obtained product was then diluted using the culture medium to get finally appropriate concentration in each well containing cells (0.25–1.5 MBq/well).

### 3.2. Characterization of Cubosomes 

**Figure 2:**
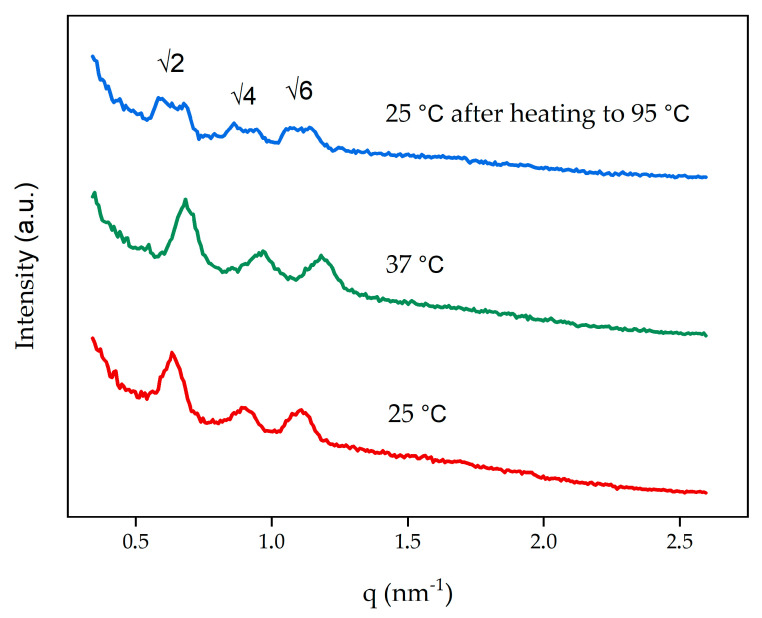
Small-angle X-ray scattering (SAXS) diffraction patterns obtained for non-doped cubosomes at 25 °C, 37 °C, and 25 °C following equilibration after heating to 95 °C.

Due to the temperatures required for DOTAGA-OA radiolabeling, we applied SAXS to assess whether 95 °C affected the cubosomes structure after cooling the dispersion to room temperature. After heating, the blank cubosome dispersion was left to cool and equilibrate at 25 °C. The SAXS measurements confirmed that the cubosomes returned to a primitive cubic phase (I$m\bar{3}$m) structure with a lattice parameter of 14.0 nm, which is the same structure when they are synthesized at 25 °C or 37 °C (Figure 2, Table 2). After cooling the system from 95 °C to 25 °C the twin peaks on diffraction patterns were present—this effect, known as supercooling was described in the literature [42]. Furthermore, the DLS measurements showed no significant changes in the size of cubosomes (214.8 ± 5 nm) or the PDI (0.271 ± 0.026), which confirmed that the homogeneity of the dispersion was retained. 

SAXS was also used to identify the type and structural parameters of cubosomes obtained in formulations containing DOTAGA-OA or DOX DOTAGA-OA.

**Figure 3:**
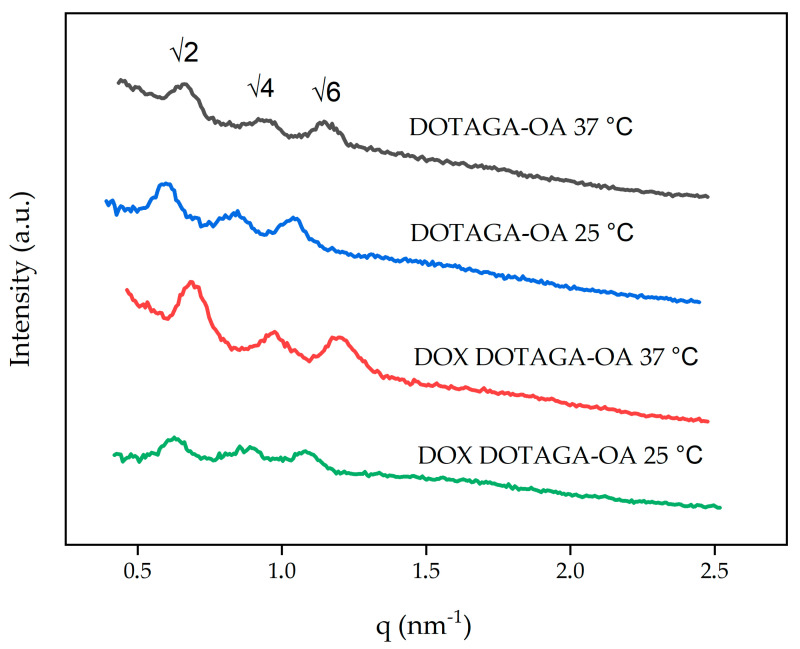
One-dimensional diffraction patterns of cubosome formulations.

Figure 3 shows the SAXS diffraction patterns obtained for the dispersed systems used in this study. Three diffraction peaks appeared in ratios of √2, √4, √6 and correspond to the primitive cubic- I$m\bar{3}$m structure [43]. 

Incorporating 0.47 wt.% DOTAGA-OA increased the lattice parameter. Doping the cubosomes with a combination of DOX and DOTAGA-OA did not significantly change the size of the unit cell and, therefore, did not affect the symmetry of the cubosomes. In 37 °C the size of the unit cell decreases for both samples. The unbound DOX was separated before measurements and encapsulation efficiency (EE (%)) was determined to be 96% ± 2%.

The physicochemical properties of cubosome formulations were characterized using dynamic light scattering (DLS). The sizes, polydispersity indexes (PDI), and zeta potentials are collected in Table 3. The particle diameter of the blank cubosomes was 181 ± 10 nm with a PDI of 0.23. For DOTAGA-OA-loaded cubosomes, the mean diameter was slightly lower, 153 ± 12 nm, with a PDI close to 0.19. In the case of cubosomes containing DOTAGA-OA and DOX, the mean diameter was approximately 160 ± 5 nm with a PDI of 0.19; showing that loading DOTAGA-OA, or DOX with DOTAGA-OA leads to a slight decrease of the cubosome size. The PDI of nanoparticles indicated a highly homogeneous particle size and also shows the stability of the structures against aggregation. 

The zeta potential of the nanoparticle formulations was approximately −27 mV for blank cubosomes. For cubosomes doped with DOTAGA-OA and DOX DOTAGA-OA, the zeta potentials were −11 mV and −19 mV (Table 3). The lower zeta potential is due to the incorporation of DOTAGA-OA ligand, which is partly removed when positively charged DOX is introduced. 

The stability of the cubosomes in time was determined using DLS (Supplementary Material Table S1). The measurements to test the stability were done immediately after the cubosomes synthesis, and after 96 h. There were no significant changes either in the zeta potentials or in PDI values, confirming that cubosomes are stable within 96 h. The zeta potential value in the beginning of the experiment was −28.7 ± 0.4 mV and after 96 h it dropped to −21.9 ± 0.8 mV. 

**Figure 4:**
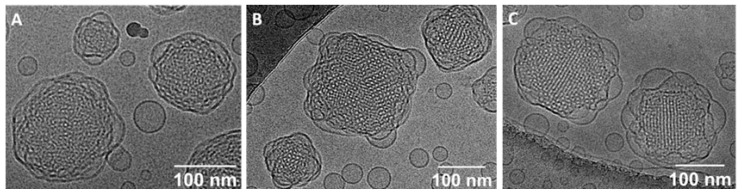
Cryogenic transmission electron microscopy (cryo-TEM) images of (**A**) blank cubosomes, (**B**) cubosomes doped with DOTAGA-OA, (**C**) doxorubicin (DOX) and DOTAGA-OA.

Cryo-TEM was used to image the obtained cubosome dispersions without changing their structure. The cryo-TEM images of blank cubosomes and cubosomes loaded with DOTAGA-OA or DOX and DOTAGA-OA revealed a well-ordered structure inside the nanoparticles (Figure 4).

### 3.3. Radiolabeling of Cubosomes Loaded with DOX and DOTAGA-OA and Carrier Stability 

Due to the kinetic inertness of DOTAGA complexes, labeling of the cubosomes with DOX and the radioconjugate had to be performed under an elevated temperature (95 °C). The labeling yield was >99%, which indicates good accessibility of DOTAGA ligand in the cubosomes. The specific activity of the obtained product was 2.5 MBq/nmol. The stability of cubosomes with DOX DOTAGA-OA-^177^Lu complex in PBS and human serum was acceptable. After in vitro incubation at 37 °C for 24 h in PBS buffer and human serum, more than 86% and 78% of radioactivity, respectively, was retained in the intact cubosome. Longer incubation than 96 h decreased the stability of radioconjugates tested in PBS (80%) as well as in human serum (65%). Due to the high stability of ^177^Lu-DOTAGA complexes, it can be assumed that ^177^Lu in form of ^177^Lu-DOTAGA-OA complex is similarly stable. 

### 3.4. Cytotoxicity Studies 

**Figure 5:**
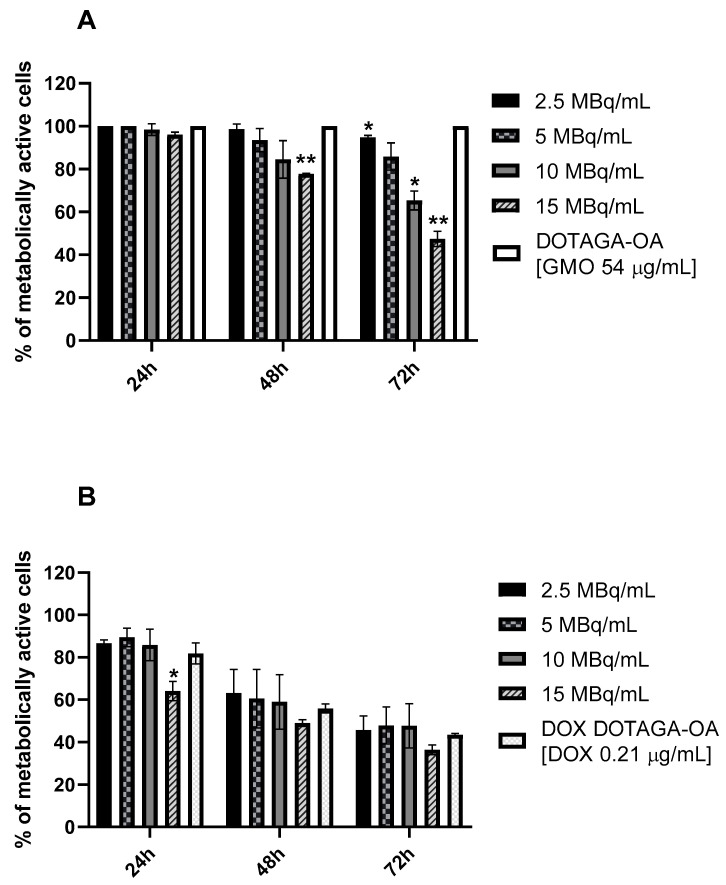
Viability of HeLa cells treated with cubosomes doped with: (**A**) DOTAGA-OA-^177^Lu and (**B**) DOX DOTAGA-OA-^177^Lu. DOTAGA-OA and DOX DOTAGA-OA cubosomes were used as a control. Data points and SD are taken from three or more measurements. Statistical significance was considered if *p* ≤ 0.05 (*) and *p* ≤ 0.01 (**).

To evaluate the anticancer activity of cubosomes doped with DOTAGA-OA-^177^Lu complex and DOX, in vitro cell cytotoxicity studies using an MTS assay were performed (Figure 5). The obtained viability results showed that cubosomes are non-toxic for HeLa cells up to a GMO concentration of 54 µg/mL (Figure 5A; Supplementary Material Figure S4). The effect of β^−^ emission from ^177^Lu on the viability of HeLa cells is also presented in Figure 5A. The cubosomes with DOTAGA-OA-^177^Lu complex decreased the metabolic activity of HeLa cells in time- and dose-dependent manners, to 47.4 ± 3.54% for the highest applied radioactivity of 15 MBq/mL and the longest incubation time of 72 h. The obtained *IC*_50_ (15 MBq/mL) is slightly higher than that obtained in similar cytotoxic studies of liposomes labeled with ^131^I on HeLa cells [44]. However, the energy of β^−^ particles emitted from ^131^I is higher than that emitted by ^177^Lu, which is important to consider when the nanoparticles do not have a vector, and because long-range β-particle penetration increases cytotoxicity. 

Doxorubicin is not affected by the acetate buffer and temperatures used (Supplementary Material Figures S5 and S6). The concentration of DOX in the cubosome, 0.21 µg/mL, was selected based on the results of our previous cytotoxicity studies performed on HeLa cells [34]. Various concentrations of DOX were used also in the present investigation (see Supplementary Material Table S2) in order to distinguish the contribution to the cell viability of the chemotherapy and β^−^radiation agents. The addition of doxorubicin to the cubosomes resulted in cytotoxicity, as described previously [34]. The *IC*_50_ determined for cubosomes loaded with DOX was ca. 0.5 µg/mL and 0.2 µg/mL when the cells were incubated at 37 °C for 48 h and 72 h, respectively (Supplementary Material Table S2). The viability of cancer cells treated with DOX (0.21 µg/msent) incorporated in the cubosomes (Figure 5B) was 43.6 ± 4.26% while the formulation of cubosomes with both DOTAGA-OA-^177^Lu and DOX showed larger cytotoxicity (36.5 ± 2.33% of the viable cells) than the cubosomes containing DOTAGA-OA ligand or DOX or DOTAGA-OA-^177^Lu complex as single drug components.

As shown in Figure 5B, the observed cytotoxic effect of the combined chemotherapeutic DOX and β-emitter ^177^Lu after 72 h of incubation is small and statistically not significant. No synergy or additivity was observed, as is usually seen in the combination of doxorubicin treatment together with external irradiation [45]. In these studies, the median survival of patients with anaplastic thyroid carcinoma (ATC) treated with low-dose weekly doxorubicin and concurrent radiation therapy was improved for six months in comparison to patients were surgery or radiation therapy were applied. 

Such a small effect observed in our experiments is due to the much stronger cytotoxicity of doxorubicin compared with β^−^ radiation (Supplementary Material Figure S7). Even the addition of high doses of ^177^Lu (15 MBq/mL) reduced cell survival only slightly. Statistically significant cytotoxicity was, however, observed at shorter incubation times, e.g., of 24 h, where the survival of cells treated with cubosomes containing 15 MBq/mL ^177^Lu and 0.21 µg/mL DOX was almost 20% lower than when using only cubosomes with 0.21 µg/mL DOX. Only small change of cytotoxicity after 48 h and 72 h when combination of DOX and radionuclide ^177^Lu was used could be ascribed to the poor stability of DOX cubosomes at long incubation times in the presence of radiation. Therefore, further investigations should include shorter life α-emitting radionuclides that deposit much more energy at shorter times. Alpha emitters such as ^213^Bi and ^211^At may be more useful for such combination therapies. 

## 4. Conclusions

This is the first study on bifunctional cubosomes containing anticancer chemotherapeutic and a radionuclide complex, and is an extension of our previous reports on monofunctional cubosomes [19,34]. The results of this study lead us to conclude that the gain in cytotoxicity achieved by combining chemotherapeutic agent, doxorubicin with the β-emitting radionuclide complex, DOTAGA-OA-^177^Lu in a single cubosomal carrier is small, and the cytotoxicity enhancement becomes statistically significant only after shorter incubation times (e.g., 24 h). Nevertheless, the multifunctional cubosomes would allow the application of smaller doses of chemotherapeutics, which significantly reduces the risk of side effects during chemotherapy. This is especially important in the case of doxorubicin, which is known to be highly cardiotoxic. Additional studies including different radionuclides are planned in order to achieve improved effectiveness of the treatment. They would include using shorter-lived radionuclides (e.g., ^211^At, ^213^Bi) to better fit to the timescale of DOX containing cubosomes in the biological environment. Studies of the addressed carriers should be also carried out in order to decrease accumulation of the drugs in liver, lungs, and spleen. Conjugation of targeting molecules would lead to more selective and efficient drug delivery to the tumor. The observed limited stability of GMO-based liquid-crystalline nanoparticles in the physiological media, prompts also to search for other lipid-like molecules to be considered as cubosome-forming materials.

Our studies open a new direction in the application of cubosomes—as the bimodal drug carriers. At the same time, this research can be considered as a step towards synthesis of combined radionuclide-chemotherapeutic drugs for a new therapeutic approach, targeted nanobrachytherapy [46].

## Figures and Tables

**Table 1:** The final compositions of the cubosome formulations.

Cubosome Formulations	Final Compositions	Components Ratio (wt.%)
Blank	buffer/GMO/F-127	94.62/4.84/0.54
DOTAGA-OA	buffer/GMO/DOTAGA-OA/F-127	94.18/4.81/0.47/0.54
DOX DOTAGA-OA	buffer/GMO/DOX/DOTAGA-OA/F-127	94.15/4.82/0.02/0.47/0.54

**Table 2:** SAXS properties of the cubosomes obtained at different temperatures.

Cubosomes Formulations	Symmetry	Unit Cell (nm)
Blank 25 °C	I$m\bar{3}$m	14.0
Blank 37 °C	I$m\bar{3}$m	13.0
Blank 25 °C (after heating to 95 °C)	I$m\bar{3}$m	14.0

**Table 3:** Properties of cubosome formulations determined using SAXS and dynamic light scattering (DLS).

Cubosome Formulations	Symmetry	Unit Cell (nm)	Size (nm)	PDI	Zeta Potential (mV)
Blank	I$m\bar{3}$m	14.0	181 ± 10	0.23 ± 0.01	−27 ± 0.9
DOTAGA-OA	I$m\bar{3}$m	14.9 ^A^ (13.5 ^B^)	153 ± 12	0.19 ± 0.03	−11 ± 0.7
DOX DOTAGA-OA	I$m\bar{3}$m	14.2 ^A^ (13.0 ^B^)	160 ± 5	0.19 ± 0.02	−19 ± 0.8

^A^ 25 °C; ^B^ 37 °C.

## Supplementary Materials

The following are available online at https://www.mdpi.com/2079-4991/10/11/2272/s1, Figure S1. ^1^H NMR spectrum of *p*-NCS-benzyl-DOTAGA-oleylamine in DMSO-*d*_6_ (298 K), Figure S2. ^13^C NMR spectrum of *p*-NCS-benzyl-DOTAGA-oleylamine in DMSO-*d*_6_ (298 K), Figure S3. ESI-MS spectrum of *p*-NCS-benzyl-DOTAGA-oleylamine, Figure S4. Viability of HeLa cells treated with cubosomes at various GMO concentrations. Non-treated cells were used as a control. Data points and SD are taken from three or more measurements. Statistical significance was considered if *p* ≤ 0.05 (*), Figure S5. UV-Vis spectra of DOX solution in 25 °C (blue line) and heated to 95 °C (measured at 25 °C following equilibration after heating—pink line), Figure S6. Viability of HeLa cells treated with acetate buffer, acetate buffer with DOX and acetate buffer with DOX heated in 95 °C. Non-treated cells were used as a control. Data points and SD are taken from three or more measurements. Statistical significance was considered if *p* ≤ 0.05 (*), *p* ≤ 0.01 (**) and *p* ≤ 0.001 (***), Figure S7. Viability of HeLa cells treated with cubosomes doped with: DOTAGA-OA-^177^Lu (^177^Lu) and DOX DOTAGA-OA-^177^Lu (^177^Lu+DOX). Data points and SD are taken from three or more measurements. Statistical significance was considered if *p* ≤ 0.05 (*), *p* ≤ 0.01 (**), *p* ≤ 0.001 (***) and *p* ≤ 0.0001 (****). Table S1. Stability of the blank cubosomes in time, Table S2. *IC*_50_ of DOX cubosomes.

## References

[1] McGowan J.V., Chung R., Maulik A., Piotrowska I., Walker J.M., Yellon D.M. (2017). Anthracycline chemotherapy and cardiotoxicity. Cardiovasc. Drugs Ther..

[2] Schmitz K.H., Prosnitz R.G., Schwartz A.L., Carver J.R. (2012). Prospective surveillance and management of cardiac toxicity and health in breast cancer survivors. Cancer.

[3] Lo S.S., Teh B.S., Jiang G.-L., Mayr N.A., Lo S.S., Teh B.S., Jiang G.-L., Mayr N.A. (2020). Controversies in Radiation Oncology.

[4] Dimanche-Boitrel M.-T., Pelletier H., Genne P., Petit J.-M., Le Grimellec C., Canal P., Ardiet C., Bastian G., Chauffert B. (1992). Confluence-dependent resistance in human colon cancer cells: Role of reduced drug accumulation and low intrinsic chemosensitivity of resting cells. Int. J. Cancer.

[5] Steel G.G., Peckham M.J. (1979). Exploitable mechanisms in combined radiotherapy-chemotherapy: The concept of additivity. Int. J. Radiat. Oncol. Biol. Phys..

[6] Nelson D.F. (1999). Radiotherapy in the treatment of primary central nervous system lymphoma (PCNSL). J. Neurooncol..

[7] Gao J., Fang L., Sun D., Shen Y., Hu Y., Li N., Chang J., Li W., Tan J. (2020). 131I-labeled and DOX-loaded multifunctional nanoliposomes for radiotherapy and chemotherapy in brain gliomas. Brain Res..

[8] Chen M.H., Chang C.H., Chang Y.J., Chen L.C., Yu C.Y., Wu Y.H., Lee W.C., Yeh C.H., Lin F.H., Lee T.W. (2010). MicroSPECT/CT imaging and pharmacokinetics of 188Re-(DXR)-liposome in human colorectal adenocarcinoma-bearing mice. Anticancer Res..

[9] Ludwig J.M., Xing M., Gai Y., Sun L., Zeng D., Kim H.S. (2017). Targeted yttrium 89-doxorubicin drug-eluting bead—A safety and feasibility pilot study in a rabbit liver cancer model. Mol. Pharm..

[10] Huang F.Y.J., Lee T.W., Chang C.H., Chen L.C., Hsu W.H., Chang C.W., Lo J.M. (2015). Evaluation of 188Re-labeled PEGylated nanoliposome as a radionuclide therapeutic agent in an orthotopic glioma-bearing rat model. Int. J. Nanomed..

[11] Hsu W.H., Liu S.Y., Chang Y.J., Chang C.H., Ting G., Lee T.W. (2014). The PEGylated liposomal doxorubicin improves the delivery and therapeutic efficiency of 188Re-Liposome by modulating phagocytosis in C26 murine colon carcinoma tumor model. Nucl. Med. Biol..

[12] Soundararajan A., Bao A., Phillips W.T., McManus L.M., Goins B.A. (2011). Chemoradionuclide therapy with 186Re-labeled liposomal doxorubicin: Toxicity, dosimetry, and therapeutic response. Cancer Biother. Radiopharm..

[13] Liu Y., Yu X.M., Sun R.J., Pan X.L. (2017). Folate-functionalized lipid nanoemulsion to deliver chemo-radiotherapeutics together for the effective treatment of nasopharyngeal carcinoma. AAPS PharmSciTech.

[14] Fong C., Le T., Drummond C.J. (2012). Lyotropic liquid crystal engineering–ordered nanostructured small molecule amphiphile self-assembly materials by design. Chem. Soc. Rev..

[15] Guo C., Wang J., Cao F., Lee R.J., Zhai G. (2010). Lyotropic liquid crystal systems in drug delivery. Drug Discov. Today.

[16] Clogston J., Caffrey M. (2005). Controlling release from the lipidic cubic phase. Amino acids, peptides, proteins and nucleic acids. J. Control. Release.

[17] Hinton T.M., Grusche F., Acharya D., Shukla R., Bansal V., Waddington L.J., Monaghan P., Muir B.W. (2014). Bicontinuous cubic phase nanoparticle lipid chemistry affects toxicity in cultured cells. Toxicol. Res..

[18] Engström S., Nordén T.P., Nyquist H. (1999). Cubic phases for studies of drug partition into lipid bilayers. Eur. J. Pharm. Sci..

[19] Mierzwa M., Cytryniak A., Krysiński P., Bilewicz R. (2019). Lipidic liquid crystalline cubic phases and magnetocubosomes as methotrexate carriers. Nanomaterials.

[20] Azmi I.D.M., Moghimi S.M., Yaghmur A. (2015). Cubosomes and hexosomes as versatile platforms for drug delivery. Ther. Deliv..

[21] Karami Z., Hamidi M. (2016). Cubosomes: Remarkable drug delivery potential. Drug Discov. Today.

[22] Angelova A., Garamus V.M., Angelov B., Tian Z., Li Y., Zou A. (2017). Advances in structural design of lipid-based nanoparticle carriers for delivery of macromolecular drugs, phytochemicals and anti-tumor agents. Adv. Colloid Interface Sci..

[23] Meli V., Caltagirone C., Sinico C., Lai F., Falchi A.M., Monduzzi M., Obiols-Rabasa M., Picci G., Rosa A., Schmidt J. (2017). Theranostic hexosomes for cancer treatments: An in vitro study. New J. Chem..

[24] Nazaruk E., Szlezak M., Górecka E., Bilewicz R., Osornio Y.M., Uebelhart P., Landau E.M. (2014). Design and assembly of pH-sensitive lipidic cubic phase matrices for drug release. Langmuir.

[25] Azhari H., Strauss M., Hook S., Boyd B.J., Rizwan S.B. (2016). Stabilising cubosomes with Tween 80 as a step towards targeting lipid nanocarriers to the blood–brain barrier. Eur. J. Pharm. Biopharm..

[26] Meli V., Caltagirone C., Falchi A.M., Hyde S.T., Lippolis V., Monduzzi M., Obiols-Rabasa M., Rosa A., Schmidt J., Talmon Y. (2015). Docetaxel-loaded fluorescent liquid-crystalline nanoparticles for cancer theranostics. Langmuir.

[27] Godlewska M., Majkowska-Pilip A., Stachurska A., Biernat J.F., Gaweł D., Nazaruk E. (2019). Voltammetric and biological studies of folate-targeted non-lamellar lipid mesophases. Electrochim. Acta.

[28] Aleandri S., Bandera D., Mezzenga R., Landau E.M. (2015). Biotinylated cubosomes: A versatile tool for active targeting and codelivery of paclitaxel and a fluorescein-based lipid dye. Langmuir.

[29] Caltagirone C., Falchi A.M., Lampis S., Lippolis V., Meli V., Monduzzi M., Prodi L., Schmidt J., Sgarzi M., Talmon Y. (2014). Cancer-cell-targeted theranostic cubosomes. Langmuir.

[30] Zhang L., Li J., Tian D., Sun L., Wang X., Tian M. (2020). Theranostic combinatorial drug-loaded coated cubosomes for enhanced targeting and efficacy against cancer cells. Cell Death Dis..

[31] Li Y., Angelova A., Hu F., Garamus V.M., Peng C., Li N., Liu J., Liu D., Zou A. (2019). PH responsiveness of hexosomes and cubosomes for combined delivery of *Brucea javanica* oil and doxorubicin. Langmuir.

[32] Liu G., Conn C.E., Waddington L.J., Mudie S.T., Drummond C.J. (2010). Colloidal amphiphile self-assembly particles composed of gadolinium oleate and myverol: Evaluation as contrast agents for magnetic resonance imaging. Langmuir.

[33] Tran N., Bye N., Moffat B.A., Wright D.K., Cuddihy A., Hinton T.M., Hawley A.M., Reynolds N.P., Waddington L.J., Mulet X. (2017). Dual-modality NIRF-MRI cubosomes and hexosomes: High throughput formulation and in vivo biodistribution. Mater. Sci. Eng. C.

[34] Nazaruk E., Majkowska-Pilip A., Bilewicz R. (2017). Lipidic cubic-phase nanoparticles-cubosomes for efficient drug delivery to cancer cells. ChemPlusChem.

[35] Barriga H.M.G., Holme M.N., Stevens M.M. (2019). Cubosomes: The next generation of smart lipid nanoparticles?. Angew. Chem. Int. Ed..

[36] Murgia S., Biffi S., Mezzenga R. (2020). Recent advances of non-lamellar lyotropic liquid crystalline nanoparticles in nanomedicine. Curr. Opin. Colloid Interface Sci..

[37] Van’t Hag L., Gras S.L., Conn C.E., Drummond C.J. (2017). Lyotropic liquid crystal engineering moving beyond binary compositional space-ordered nanostructured amphiphile self-assembly materials by design. Chem. Soc. Rev..

[38] Mertins O., Mathews P.D., Angelova A. (2020). Advances in the design of ph-sensitive cubosome liquid crystalline nanocarriers for drug delivery applications. Nanomaterials.

[39] Nazaruk E., Majkowska-Pilip A., Godlewska M., Salamończyk M., Gawel D. (2018). Electrochemical and biological characterization of lyotropic liquid crystalline phases—Retardation of drug release from hexagonal mesophases. J. Electroanal. Chem..

[40] Majkowska-Pilip A., Kozminski P., Wawrzynowska A., Budlewski T., Kostkiewicz B., Gniazdowska E. (2018). Application of neurokinin-1 receptor in targeted strategies for glioma treatment. Part I: Synthesis and evaluation of substance P fragments labeled with 99mTc and 177Lu as potential receptor radiopharmaceuticals. Molecules.

[41] Nazaruk E., Miszta P., Filipek S., Górecka E., Landau E.M., Bilewicz R. (2015). Lyotropic cubic phases for drug delivery: Diffusion and sustained release from the mesophase evaluated by electrochemical methods. Langmuir.

[42] Dong Y.D., Tilley A.J., Larson I., Lawrence M.J., Amenitsch H., Rappolt M., Hanley T., Boyd B.J. (2010). Nonequilibrium effects in self-assembled mesophase materials: Unexpected supercooling effects for cubosomes and hexosomes. Langmuir.

[43] Hartnett T.E., Ladewig K., O’Connor A.J., Hartley P.G., McLean K.M. (2015). Physicochemical and cytotoxicity analysis of glycerol monoolein-based nanoparticles. RSC Adv..

[44] Li W., Sun D., Li N., Shen Y., Hu Y., Tan J. (2018). Therapy of cervical cancer using 131 I-labeled nanoparticles. J. Int. Med. Res..

[45] Sherman E.J., Lim S.H., Ho A.L., Ghossein R.A., Fury M.G., Shaha A.R., Rivera M., Lin O., Wolden S., Lee N.Y. (2011). Concurrent doxorubicin and radiotherapy for anaplastic thyroid cancer: A critical re-evaluation including uniform pathologic review. Radiother. Oncol..

[46] Cai Z., Yook S., Lu Y., Bergstrom D., Winnik M.A., Pignol J.P., Reilly R.M. (2017). Local radiation treatment of HER2-positive breast cancer using trastuzumab-modified gold nanoparticles labeled with 177Lu. Pharm. Res..

