# Supplementary material for: Lipidic Cubic-Phase Nanoparticles (Cubosomes) Loaded with Doxorubicin and Labeled with 177Lu as a Potential Tool for Combined Chemo and Internal Radiotherapy for Cancers
Source: Nanomaterials (Basel). 2020 Nov 16;10(11):2272. doi: 10.3390/nano10112272 (PMC7696353; doi:10.3390/nano10112272)
Supplement: Supplementary file 1 [file nanomaterials-10-02272-s001.pdf]

nanomaterials

Supplementary Material

Lipidic Cubic-Phase Nanoparticles (Cubosomes) Loaded with Doxorubicin and Labeled with ^{177}Lu as a Potential Tool for Combined Chemo and Internal Radiotherapy for Cancers

Adrianna Cytryniak [1], Ewa Nazaruk [1], Renata Bilewicz [1], Emilia Górzyńska [2], Kinga Żelechowska-Matysiak [2], Rafał Walczak [2], Adam Mames [3], Aleksander Bilewicz [2] and Agnieszka Majkowska-Pilip [2,*]

1 Faculty of Chemistry, University of Warsaw, Pasteura 1 St., 02-093 Warsaw, Poland; acytryniak@chem.uw.edu.pl (A.C.); enaz@chem.uw.edu.pl (E.N.); bilewicz@chem.uw.edu.pl (R.B.)
2 Centre of Radiochemistry and Nuclear Chemistry, Institute of Nuclear Chemistry and Technology, Dorodna 16 St., 03-195 Warsaw, Poland; e.gorzynska@student.uw.edu.pl (E.G.); k.zelechowska@ichtj.waw.pl (K.Ż.-M.); r.walczak@ichtj.waw.pl (R.W.); a.bilewicz@ichtj.waw.pl (A.B.)
3 Institute of Physical Chemistry, Polish Academy of Sciences, Kasprzaka 44/52 St., 01-224 Warsaw, Poland; amames@ichf.edu.pl
* Correspondence: a.majkowska@ichtj.waw.pl; Tel.: +48-22-504-1011

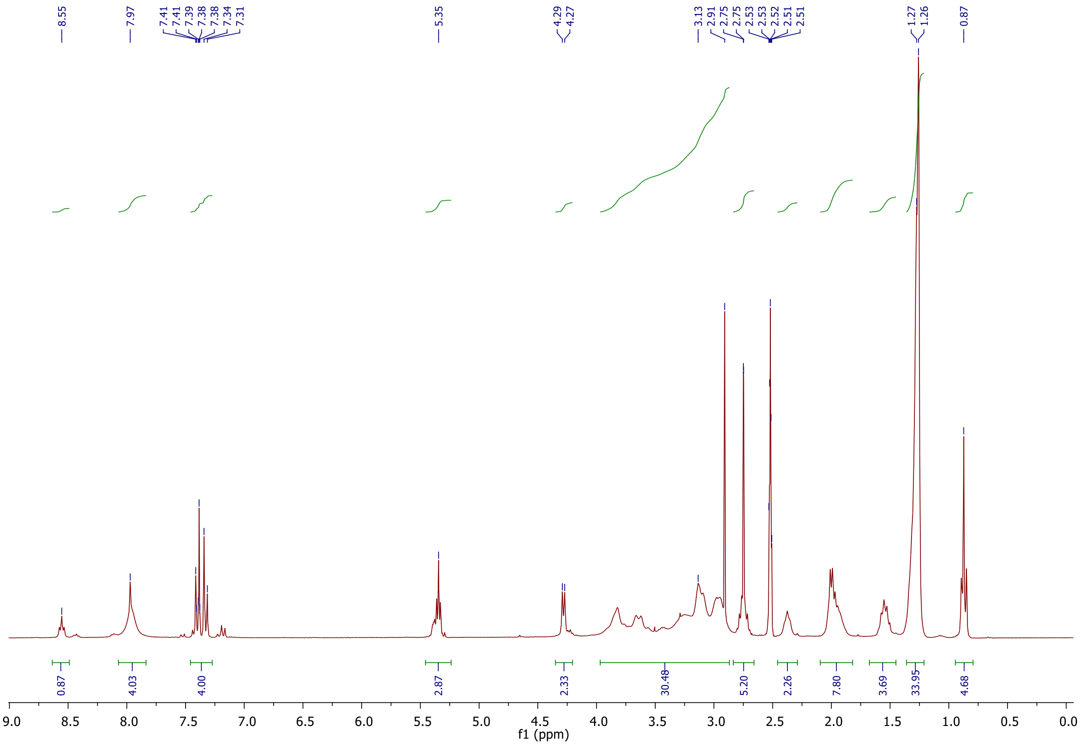

Figure S1. ^{1}H NMR spectrum of *p*-NCS-benzyl-DOTAGA-oleylamine in DMSO-*d6* (298 K).

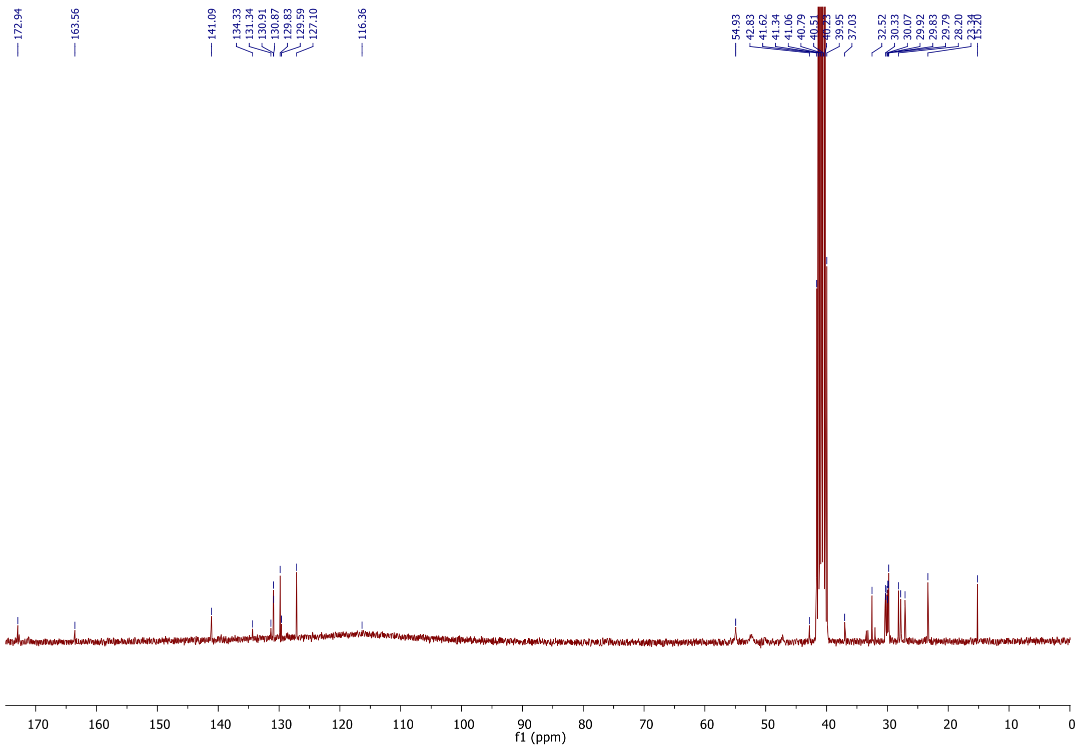

Figure S2. ^{13}C NMR spectrum of *p*-NCS-benzyl-DOTAGA-oleylamine in DMSO-*d6* (298 K).

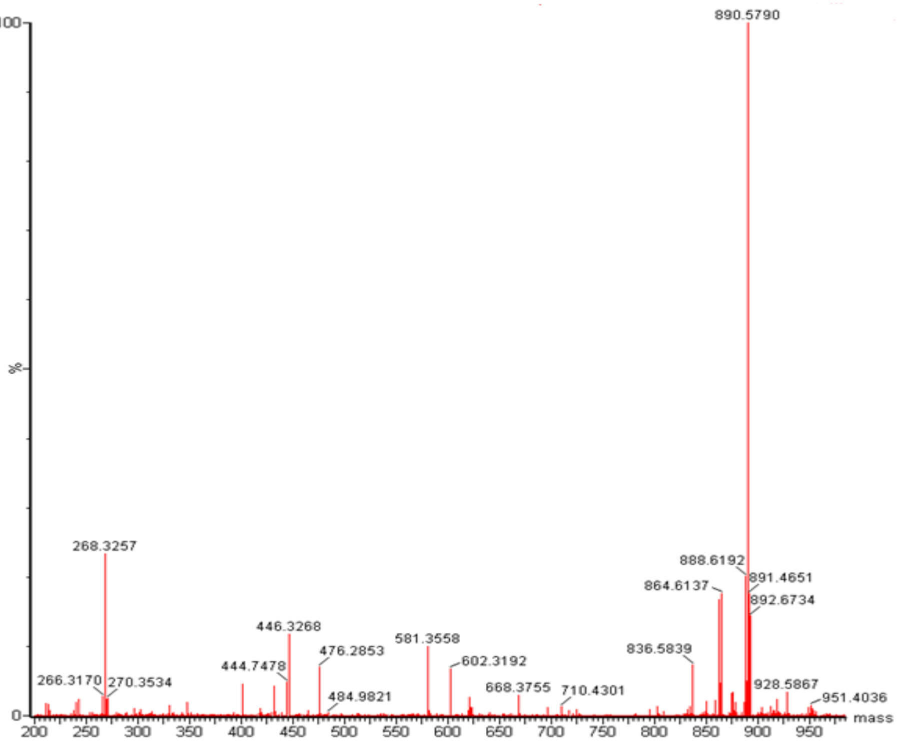

Figure S3. ESI-MS spectrum of *p*-NCS-benzyl-DOTAGA-oleylamine.

Table S1. Stability of the blank cubosomes in time.

Time (h)	PDI	Zeta Potential (mV)
0	0.16 ± 0.03	−28.7 ± 0.4
8	0.19 ± 0.01	−25.9 ± 1.3
24	0.19 ± 0.01	−23.5 ± 1.2
48	0.16 ± 0.02	−22.9 ± 1.1
72	0.21 ± 0.01	−23.3 ± 1.3
96	0.23 ± 0.01	−21.9 ± 0.8

Table S2. IC_{50} of DOX cubosomes.

DOX Cubosomes	
**Incubation Time**	**IC_{50} (μg/mL)**
24 h	1.26 ± 0.33
48 h	0.51 ± 0.14
72 h	0.20 ± 0.09

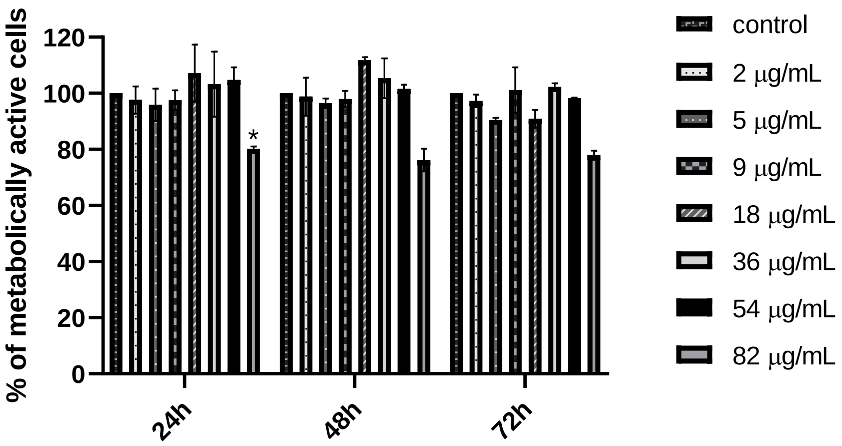

Figure S4. Viability of HeLa cells treated with cubosomes at various GMO concentrations. Non-treated cells were used as a control. Data points and SD are taken from three or more measurements. Statistical significance was considered if $p \leq 0.05$ (*).

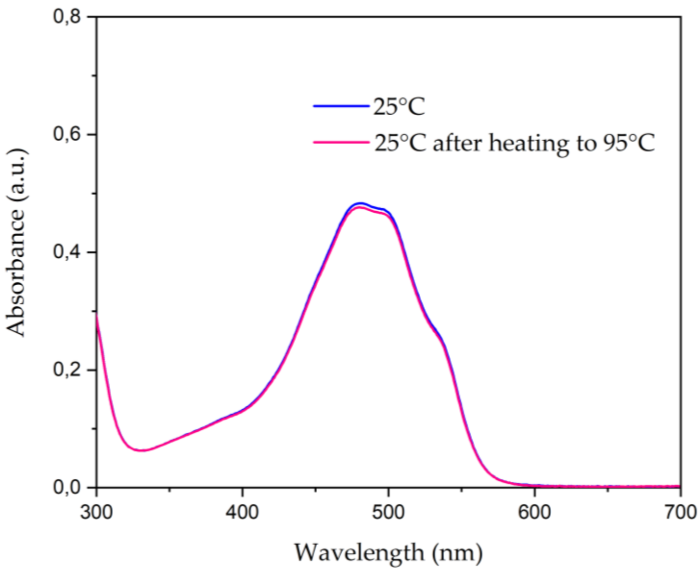

Figure S5. UV-Vis spectra of DOX solution in 25 °C (blue line) and heated to 95 °C (measured at 25 °C following equilibration after heating – pink line).

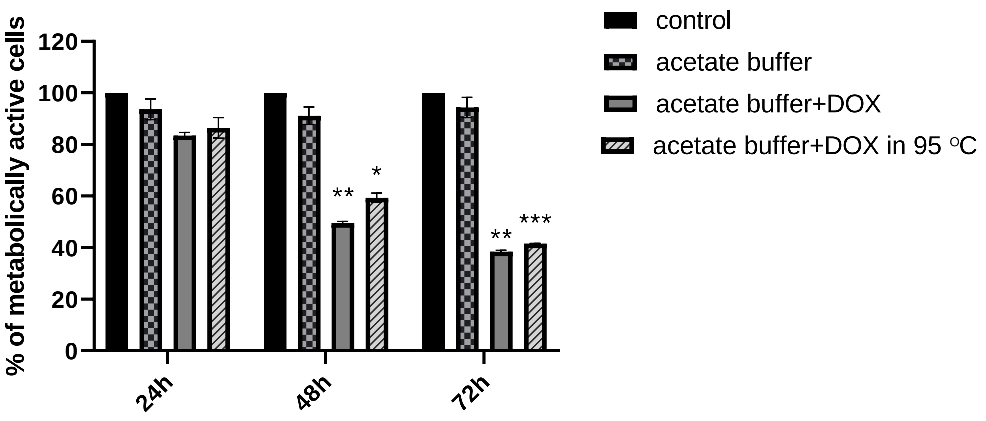

Figure S6. Viability of HeLa cells treated with acetate buffer, acetate buffer with DOX and acetate buffer with DOX heated in 95 °C. Non-treated cells were used as a control. Data points and SD are taken from three or more measurements. Statistical significance was considered if $p \leq 0.05$ (*), $p \leq 0.01$ (**) and $p \leq 0.001$ (***).

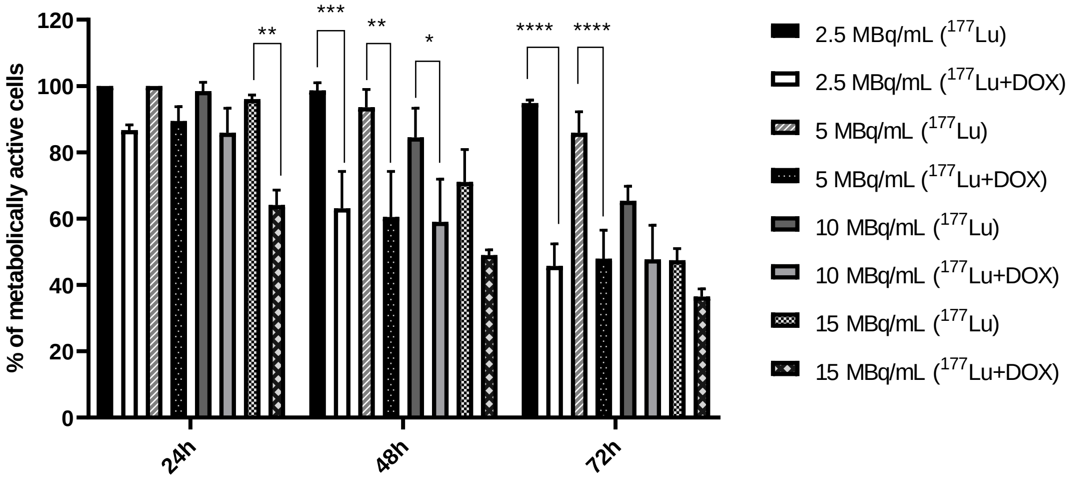

Figure S7. Viability of HeLa cells treated with cubosomes doped with: DOTAGA-OA-^{177}Lu (^{177}Lu) and DOX DOTAGA-OA-^{177}Lu (^{177}Lu + DOX). Data points and SD are taken from three or more measurements. Statistical significance was considered if $p \leq 0.05$ (*), $p \leq 0.01$ (**), $p \leq 0.001$ (***) and $p \leq 0.0001$ (****).

© 2020 by the authors. Licensee MDPI, Basel, Switzerland. This article is an open access article distributed under the terms and conditions of the Creative Commons Attribution (CC BY) license (http://creativecommons.org/licenses/by/4.0/).
